# Home/Community-Based Medical and Elderly Care Services Utilization in China: A Cross-Sectional Study from the Middle-Aged and Elderly Population

**DOI:** 10.3390/healthcare11172431

**Published:** 2023-08-30

**Authors:** Shangren Qin, Yenuan Cheng, Hangjing Zhang, Ye Ding

**Affiliations:** 1 School of Public Health, Hangzhou Normal University, Hangzhou 311121, China; qinshangren@hznu.edu.cn (S.Q.); chengyenuan@stu.hznu.edu.cn (Y.C.); zhanghangjing@stu.hznu.edu.cn (H.Z.); 2 School of Public Health, Hangzhou Medical College, Hangzhou 311399, China

**Keywords:** medical services, elderly care services, physical examination, onsite visit, health management, entertainment, elderly, China

## Abstract

Few studies have analyzed the acceptance of home/community-based medical and elderly care services in China. Therefore, we conducted a cross-sectional study to describe the acceptance of five services among people aged ≥ 45 years in the China mainland, and their influencing factors. The data were obtained from the database China Health and Retirement Longitudinal Study 2018. For each service, a binary logistics regression was adopted. A total of 9719 people were included, of whom 20.12% received services. The numbers of recipients (acceptance rates) of the five services, namely, comprehensive aged care services, regular physical examinations, onsite visits, health management, and entertainment, were 107 (1.10%), 1640 (16.87%), 323 (3.32%), 156 (1.61%), and 245 (2.52%), respectively. About 4% of people had received two or more services. The elderly aged 65–74 and those who were satisfied with the local medical services had higher acceptance of services. Urban hukou having health insurance, two or more chronic diseases, provincial economic welfare, and social welfare were positively associated with the acceptance of regular physical examination services. It is suggested that the government should gradually improve satisfaction with local medical services, and pay more attention to the needs of elderly people aged 65–74 for all kinds of home/community-based medical and elderly care services.

## 1. Introduction

The aging of the population has become a challenge for many countries. As a populous country, China’s aging problem is increasingly serious. According to the seventh census of the China National Bureau of Statistics, by 2020, there were 264 million people over 60 years old in China, accounting for 18.70% of the total population, up 5.44% compared with the sixth census in 2010 [1]. The aging problem in China has the characteristics of a large population base, rapid development, and large differences between urban and rural areas. How to deal with aging in the vast Chinese mainland is a challenge. Moreover, most elderly only have one child in China (one-child policy). Whether these elderly people receive adequate care services is also of concern.

With the deepening population aging, the elderly have raised higher demands for medical and elderly care services, which has attracted more attention from the government. China put forward the concept of “medical and elderly care services” in 2013, which is later than other countries. Other countries already have well-established models for medical and elderly care services (e.g., the PACE model in the USA). China, however, is still in the exploratory phase. In recent years, China has been continuously promoting its medical and elderly care services policy. According to the Outline of “Healthy China 2030”, China will continuously promote healthy aging, improve the combination of medical care and nursing care, and provide the elderly with medical and elderly care services integrating hospitalization during treatment, rehabilitation care, stable life care, and hospice care [2]. Based on the statistics of the National Health Commission, by 2021, there were 6492 medical and elderly care institutions in China, with an increase of 10.8% over the previous year [3]. This is the result of the government’s encouragement for social forces to set up medical and elderly care institutions.

Medical and elderly care services integrate medical and elderly care resources. Combining professional medical services (regular visits, diagnosis and treatment of diseases, etc.) with elderly care services (life care and psychological comfort), medical and care services can meet the health needs of the elderly at different stages in the nursing process [4]. That means that the elderly have access to both services simultaneously in the community, at home, or in a specialized healthcare institution. The scope of medical and elderly care services is extensive. For example, “long-term care service” is one of the medical and elderly care services. Moreover, there are many models of medical and elderly care services depending on the source of the services provided. For instance, we called the service model “Home/Community model” if the medical and elderly care services were mainly provided by families, the community, and family doctors [5].

Many scholars have studied the topic of medical and elderly care services. Relevant studies have analyzed the needs of medical and elderly care modes for the elderly. About 54% of Australian elderly women choose to receive care services from family and community [6]. A survey of the elderly in China found that 86.37% of the elderly choose to live at home. However, with the increase in the degree of disability, the elderly are more likely to choose institutional care [7]. In a survey on the preference of medical and elderly care models for the elderly in Shandong Province, China, researchers found that 89.1% of the elderly preferred family care, and 2.7% chose family–community pension. Their preference is mainly influenced by self-care ability and loneliness [8]. To sum up, it is not difficult to find that most Chinese elderly people are more inclined to receive elderly care services at home.

Some studies have analyzed the demand preference of medical and elderly care services for the elderly, finding that the rural elderly mainly prefer personal care, medical care, psychological counseling, and health education, and the elderly with a higher degree of disability are more inclined to choose medical care, health examinations, health education, etc. [9]. Elderly women suffering from various diseases or disabilities are more willing to receive long-term care services [10]. The older elderly usually have higher expectations for onsite visits and health education, and the older people with stronger loneliness are more inclined to accept onsite visits, psychological counseling, social entertainment activities, and health education services [11]. A survey of the annual physical examination of the elderly in rural areas in China found that the probability of annual physical examinations for civil servants/retirees is 2.16 times that for farmers. The elderly with higher health knowledge levels generally are more likely to receive annual physical examinations [12]. The annual physical examination rate of the rural elderly is significantly lower than that of the urban elderly. Education level, the number of non-communicable chronic diseases, self-economic status, and health insurance are the main factors that affect the acceptance of the annual physical examination of the elderly [13]. In addition, a framework of entertainment service systems for the elderly has been proposed according to their entertainment needs [14].

The above studies analyze the demand willingness and choice preference of medical and elderly care services for the elderly. However, few studies focus on the acceptance of specific items of various medical and elderly care services. Therefore, the following questions arise. What are the acceptance rates of medical and elderly care services among middle-aged and elderly people in China? What factors influence the acceptance rate? In the above studies on the demand willingness and choice preference of medical and elderly care services, Andersen’s Behavioral Model-Related Variables (e.g., sex [10], chronic diseases [13]) were the important influencing factors. Moreover, lifestyle factors (e.g., smoking, drinking) [15], satisfaction with local healthcare [16], and social environment [17] can affect people’s use of health services. Are these also factors that affect the acceptance rate of medical and elderly care services?

Therefore, we conducted this study for the following reasons. First, China’s aging challenges are great, and many elderly people have only one child. Whether the elderly receive adequate care services is of concern. Second, compared with other countries, China’s medical and elderly care service policy started late. There is still room for improvement in the supply of types of medical and elderly services provided. Third, although many scholars explored the demand willingness and choice preference of medical and elderly care services, few of them studied the acceptance rate and its influencing factors.

In this study, we aimed to describe the acceptance rates of five medical and elderly care services among people aged ≥ 45 years in the China mainland, and also explore the influencing factors. By summarizing the acceptance rates of various medical and elderly care services and comparing the differences in influencing factors of various services, we can observe which group of middle-aged and elderly people in China have accepted or consumed medical and care services, and what is the difference, in order to put forward some suggestions for improvement. In our paper, home/community-based medical and elderly care services mean that the “medical and elderly care services were mainly provided by families, community and family doctors” and it also means that the elderly can accept these services at home or in the community, including comprehensive aged care services, regular physical examinations, onsite visits, health management services, and entertainment activities.

## 2. Materials and Methods

### 2.1. Study Design and Data

The data were obtained from the 2018 survey data of CHARLS—China Health and Retirement Longitudinal Study, a public database, and the website is http://charls.pku.edu.cn/en/ (accessed on 1 August 2023). The CHARLS National Baseline Survey was launched in 2011, followed up every two to three years, aiming at collecting high-quality microscopic data of families and individuals aged 45 and above in China. The survey covered 150 county-level units, 450 village-level units, and 19,000 people in about 12,400 households in the China mainland. The CHARLS data are published publicly with high academic recognition [18].

A cross-sectional study was designed to explore the acceptance of various medical and elderly care services for middle-aged and elderly people in China and their influencing factors. Firstly, by reviewing previous literature, this study includes different kinds of variables as potential influencing factors (independent variables), including Andersen’s behavioral model-related variables (gender, age, income, etc.), lifestyle variables, and social, economic, and medical welfare variables at the provincial level. Secondly, medical and elderly care services for middle-aged and elderly people are divided into five categories: comprehensive aged care services, regular physical examinations, onsite visits, health management services, and entertainment activities. Finally, binary logistics regression models are used to explore the influencing factors of medical and elderly care services for middle-aged and elderly people.

The sample size in CHARLS 2018 was 19,816. Among them, 255 respondents were younger than 45 years and were therefore deleted. Moreover, some variables are missing in the database. For example, only 11,021 have answered the questions about the acceptance of five home/community medical and elderly care services. Finally, there are only 9719 individuals with complete information and these are included in our study.

### 2.2. Variable Measurement

#### 2.2.1. Acceptance of Medical and Care Service Items (Dependent Variable)

The questions and content options in the CHARLS questionnaire about the acceptance of home/community medical and elderly care services are as follows: “Have you ever received the following home and community care services? (circle all that apply) 1. Day care centers, nursing homes, senior dining tables, etc.; 2. Regular physical examination; 3. Onsite visits; 4. Family beds; 5. Community nursing; 6. Health management; 7. Entertainment”. Family beds are designed to use the family as a place of care, allowing patients to receive medical treatment and care in a familiar environment. Entertainment, in our opinion, is regarded as a mental health service. It could be using video game products or playing cards to improve the physical and mental health of the elderly. The contents of medical and elderly care services were divided into five categories: The first category is comprehensive aged care services (options 1, 4, and 5). Because “4. Family beds” and “5. Community nursing” were selected by a small number of people, and they can be included under old-age care as a whole with option 1, they are classified into one category. The second category is “Regular physical examination” (option 2), the third category is “Onsite visits” (option 3), the fourth category is “Health management” (option 6), and the fifth category is “Entertainment” (option 7).

#### 2.2.2. Andersen’s Behavioral Model-Related Variables (Independent Variables)

The Andersen model is one of the classical theoretical models to study the use of health services, which includes three types of factors: “predisposing-enabling-demand” [19]. Predisposing factors represent the tendency to use health services, including age, gender, marital status, household registration, and education level. In China, hukou is divided into urban hukou and rural hukou, which is information registered by individuals according to their type of residence, and each person can only register one place. Enabling factors include personal income, health insurance, and pension. In this study, personal income was calculated by questionnaire, and the sum of personal salary, retirement salary, and all subsidies or benefits (such as family planning allowance for the elderly, unemployment allowance, etc.) was divided into three groups according to the tertiles: the low-income group, the middle-income group, and the high-income group. Demand factors represent the patient’s demand characteristics, including self-reported general health status and chronic illness in this article. Self-reported general health status was divided into three groups: bad, fair, and very good/good. Chronic illness was also divided into three groups: no chronic disease, one chronic disease, and two or more chronic diseases. The values for variables are shown in each table.

#### 2.2.3. Lifestyle and Satisfaction Variables (Independent Variables)

Because people’s health is affected by many kinds of factors, in addition to genetic factors, personal lifestyle is an important direction of health intervention. Lifestyle is also associated with health service use [15]. Therefore, lifestyle variables (such as smoking and drinking) are included. The smoking variable was classified as current smokers and non-smokers. The respondents who had already quit smoking were identified as non-smokers. Moreover, based on the answer to the question “Have you ever consumed any alcoholic beverages, such as beer, wine, or spirits, in the past year?”, the drinking variable was classified as current drinkers and non-drinkers.

Relevant studies show that the lower the satisfaction with local healthcare, the lower the utilization rate of local rural health services [16]. Therefore, satisfaction with the quality, cost, and convenience of local medical services was added to our independent variables. Satisfaction was divided into three groups: satisfied (those who answered “Very satisfied” or “Somewhat satisfied”), neutral (those who answered “neutral”), and dissatisfied (those who answered “Somewhat dissatisfied” or “Very dissatisfied”).

#### 2.2.4. Provincial Social and Economic Welfare Variables (Independent Variables)

Studies have shown that social environment, like social welfare, can affect people’s use of health services [17]. There is a paper exploring the association between province-level socioeconomic welfare and depression of the Chinese elderly with the 2018 CHARLS data [20]. Three socioeconomic welfare factors (named economic welfare, social welfare, and medical welfare) are extracted from 14 province-level variables using the principal component analysis (PCA) method. The 14 province-level variables are extracted from the China Civil Affairs Statistical Yearbook for 28 provinces (the same provinces as in the CHARLS study). As the three socioeconomic welfare factors are publicly published in that article, they are used as independent variables in our study. The detailed contexts of the three welfare factors and the statistical results can be found in the original article [20].

### 2.3. Statistical Analysis

First of all, the current situations of the number, items, and rates of acceptance of various medical and nursing services among middle-aged and elderly people in China were described and compared, and the distribution differences in the number and rates of acceptance of medical and elderly care services among different groups were analyzed by using the χ^2^ test. Specifically, we calculated the acceptance rate for each service (rate = number of recipients/sample size). The sample size was 9719. For example, 107 received the comprehensive aged care service, so the number of people who did not receive this service is 9719 − 107 = 9612, and the acceptance rate was 107/9719 = 1.10%.

Second, the multicollinearity among independent variables was tested, and the results showed that the variance inflation factors (VIF) were all less than 10, indicating that there was no collinearity among independent variables. Finally, for each service, a binary logistic regression model was used to explore and compare the influencing factors. For provincial-level variables, during the statistical analysis process, we matched each case with its corresponding provincial variables, and then incorporated them into the analysis model.

All statistical tests are two-tailed, and *p* < 0.05 is considered to be statistically significant. The software is STATA 14.0 (STATA Corp., College Station, TX, USA).

## 3. Results

A total of 9719 middle-aged and elderly people aged 45 and above are included in this study. About half of them were females (4932, 50.75%), while the dominant age group was 65–74 years (4699, 48.35%). The rural population accounted for 76.99% (7483). Most of the respondents were married (7746, 79.70%), and had an elementary school education (4375, 45.01%).

The number of recipients (acceptance rates) of the five major services, namely, comprehensive aged care services, regular physical examinations, onsite visits, health management services, and entertainment activities, are 107 (1.10%), 1640 (16.87%), 323 (3.32%), 156 (1.61%), and 245 (2.52%), respectively.

### 3.1. Acceptance Rate of Medical and Elderly Care Services among Middle-Aged and Elderly People

Firstly, as shown in Table 1, there are differences in the acceptance rate of comprehensive aged care services in terms of age and marital status (*p* < 0.05). The acceptance rate increases with age. However, compared with people with other marital statuses (1.57%), the acceptance rate of married people to services is lower (0.98%).

Secondly, people aged 65–74, with urban hukou, elementary education, high income, and health insurance, who are non-smoking, have a chronic disease, and are more satisfied with local medical services, have a higher proportion of receiving regular physical examination services (*p* < 0.05). Among them, the acceptance rate of physical examination services for urban residents and the elderly aged 65–74 exceeded 20%.

Thirdly, the acceptance rate of onsite visits increases with age, and there are differences in education, income, satisfaction with local medical services, and chronic diseases (*p* < 0.05).

In addition, it can be found that the acceptance rate for health management services increases with age (*p* = 0.005). Furthermore, the acceptance rate of health management services increases with education level, income, self-rated health status, satisfaction with local medical services, degree of drinking, etc. (all *p* values < 0.05).

Finally, it can be found that there is no difference in the distribution of the acceptance rate of entertainment services among different ages. However, the acceptance rate of entertainment services increases with education level, income, self-rated health status, satisfaction with local medical services, degree of drinking, etc. (all *p* values < 0.05). The elderly with urban hukou and a pension have higher acceptance rates of entertainment services (*p* = 0.001 and 0.020).

### 3.2. Accepted Quantity and Item Type of Medical and Elderly Care Services

Among the 9719 middle-aged and elderly people included in this study, 7764 (79.88%) did not receive any medical services and 1955 (20.12%) received at least one service. The numbers of people who received one, two, or three or more services were 1566 (16.11%), 291 (2.99%), and 98 (1.01%), respectively. The distribution of service acceptance is shown in Table 2. Generally speaking, the middle-aged and elderly who are urban residents, who have health insurance, who are non-smokers, who are more satisfied with local medical services, and who have more chronic diseases are more likely to receive medical and elderly care services (*p* < 0.05). There are also differences in the distribution of the number of medical services received by middle-aged and elderly people among education levels and incomes. For example, more middle-aged and elderly people with an elementary-school education level and a middle income receive two services (*p* < 0.001).

In addition to the number of services received, this study also analyzed the types of items received (Table 3). It can be found that “2-Regular physical examination” is the most popular service for the middle-aged and elderly. In addition, middle-aged and elderly people who receive two or more medical and elderly care services choose the following item combination the most: 2 + 3 (the frequency of 2 + 3 in all kinds of combinations is the highest), that is, “Regular physical examination + Onsite visit”. The frequency of “2 + 5”, that is, “Regular physical examination + Entertainment” services is also quite high in various combinations.

### 3.3. Influencing Factors of Middle-Aged and Elderly People’s Acceptance of Medical and Elderly Care Services

The binary logistics regression results are shown in Table 4. In terms of comprehensive aged care services, the older middle-aged and elderly people who are satisfied with local medical services are more willing to accept comprehensive aged care services.

In terms of physical examination services, people aged ≥65 years with urban hukou, health insurance, high satisfaction with local medical services, and two or more chronic diseases are more willing to receive regular physical examinations (OR > 1). In addition, the acceptance of regular physical examination services for middle-aged and elderly people is positively correlated with provincial economic welfare and social welfare (OR = 1.13, 95% CI: 1.03~1.25; OR = 1.10, 95% CI: 1.03~1.17), but negatively correlated with medical welfare (OR = 0.77, 95% CI: 0.71~0.85).

As for onsite visit services, people aged ≥65 years having higher satisfaction with local medical services and more than two chronic diseases are more willing to accept onsite visit services.

For health management services, people aged ≥65 years having better self-health evaluations and higher satisfaction with local medical services show a higher probability of receiving health management services (OR > 1).

In terms of entertainment services, the elderly aged 65–74 are more likely to receive entertainment services than those aged 45–64 (OR = 1.36, 95% CI: 1.01~1.85). High education levels, good self-health evaluations, high satisfaction with local medical services, and drinking are positive influencing factors for middle-aged and elderly people receiving entertainment services (OR > 1).

## 4. Discussion

On the whole, with the increase in age, the elderly’s demand for home/community-based medical and elderly care services increases. The results are similar to those of Kathrin Steinbeisser [10], Albert Wong [21], Chen-Yi Wu [22], and Shiou-Liang Wee [23]. This shows that age has an important influence on the acceptance of all kinds of medical and elderly care services for the middle-aged and elderly.

### 4.1. Influencing Factors of Acceptance of Various Medical and Elderly Care Services

Regular physical examinations are an important medical service. This study found that urban household registration, high satisfaction with local medical services, two or more chronic diseases, health insurance, and better local economic and social welfare can encourage the middle-aged and elderly to receive regular physical examinations. Moreover, there is a positive relationship between the increase in age and the high physical examination rate, which is similar to relevant study results [24]. With the increase in age, the health of the elderly is affected by more risk factors. Regular physical examinations are an effective way to monitor and manage non-communicable diseases, and they can enhance the survival probability of the elderly [25]. Therefore, the enthusiasm of the elderly for regular physical examinations is increasing with age. Consistent with the related literature [13], this study also found that urban people are more likely to receive regular physical examinations than rural people. This phenomenon may be caused by the following factors: 1. The accessibility of regular physical examination services in rural areas is lower than that in urban areas. Although China provides regular free physical examination services for some rural elderly people, the final consumption probability is low because of the long distance and the inconvenience of traffic [26]. 2. Some rural elderly people have a great fear of discovering potentially serious diseases [13], so they may be reluctant to receive regular physical examinations. 3. Compared with the urban elderly, the rural elderly have a weak education level and health awareness, and have not yet formed the habit of regular physical examinations. 4. Most urban elderly can enjoy regular physical examinations after they retire. In rural areas, although township health institutions will be obliged to remind the elderly to have an annual physical examination, it is difficult to remind them regularly and repeatedly [12], and the participation rate is low.

In the meanwhile, the higher the evaluation of local medical services, the more likely that middle-aged and elderly people will receive regular physical examination services. Relevant studies show that the lower the satisfaction with local healthcare, the lower the utilization rate of local rural health services will be [16], and the utilization rate of medical services is positively correlated with physical examinations [27]. In addition, this study found that the middle-aged and elderly with various chronic diseases are more likely to receive regular physical examinations, which is consistent with the results of the annual physical examination survey of Chinese elderly in Canada [28]. This may be related to the increased perceived risk of the elderly. With the increase in chronic diseases, the incidence of complications and other serious and dangerous diseases increases. Therefore, the elderly must have regular physical examinations to achieve the goal of early detection, diagnosis, and treatment. In addition, health insurance and better provincial economic and social welfare are the promoting factors for the middle-aged and elderly to receive regular physical examinations. Therefore, the government can further increase the reimbursement ratio of routine regular physical examination items for the elderly, and expand the coverage of health insurance reimbursement to some extent, in order to reduce their economic burden.

The elderly aged ≥ 65 years with two or more chronic diseases have higher acceptance rates of onsite visit services. Relevant studies also found that the older elderly have a higher expected demand for onsite visits [11], which is consistent with the results of this study. Previous studies have pointed out that the elderly with higher education levels have a greater demand for on-call medical services [29]. However, this study has not found a significant influence of education level on house-visit services. In addition, the middle-aged and elderly with more chronic diseases have a higher demand for onsite visits, which may be related to their need to improve future life quality. It is found that onsite visits have a positive effect on improving the self-care efficacy of the elderly [30]. In a study on the effectiveness of preventive primary care outreach interventions aimed at older people, onsite visits were found to help reduce the mortality rate [31]. Onsite visits can positively influence the health and happiness of the elderly [32]. Therefore, by receiving regular onsite visits, the elderly can increase their preventive knowledge of physical health and maintain their quality of life.

The elderly aged ≥ 65 years have a higher acceptance of health management services, which may be related to the positive prediction of their health and strong belief in health. With the increase of age, the normal function of the body gradually declines, and the risk factors increase. With positive predictions of future physical conditions, the elderly have a higher demand for health management services to maintain a good physical condition. The elderly with better self-reported health may have higher health beliefs and be more eager to keep in good health. Study results show that the elderly over 80 years old have a high demand for health education services [11]. In addition, community health management services can improve the health status of the elderly [33]. For example, community health management has a positive impact on the long-term health status of elderly patients with type 2 diabetes [34]. Therefore, older people with better self-evaluations of their health are more inclined to use health management services to ensure a better physical condition and higher quality of life in the future.

The middle-aged and elderly who have higher education levels, better self-health evaluations, and drinking habits have higher acceptance rates of entertainment activity services. The higher the education level, the stronger the demand for entertainment activities. This may be because the middle-aged and elderly with higher education levels have wider knowledge and a higher pursuit of spiritual entertainment. In addition, health factors are significantly related to the demand for entertainment activities of the elderly. The middle-aged and elderly with poor self-evaluations of their health may decline their activity ability due to the deterioration of physical function and the influence of disease. Therefore, they accept fewer entertainment activities. Entertainment activities are related to the mental health and happy life of the elderly [14], and play a positive role in promoting their physical health, which further enhances the enthusiasm of the elderly to participate in entertainment activities. Older people with drinking habits may receive more entertainment services because they have more social activities.

### 4.2. Comparative Analysis

In particular, this study found that the elderly aged 65–74 who are highly satisfied with local medical services have a high degree of acceptance of various medical and elderly care services such as old-age care services, regular physical examinations, onsite visits, health management, and entertainment activities. This result may have a certain predictive effect on the future demand for aged care services, suggesting that medical service institutions, government, and other relevant departments should pay attention to the improvement of the above-mentioned service content and quality. According to the data of the National Bureau of Statistics of the People’s Republic of China, the number of people aged 65–74 is the largest among all ages over 60. Therefore, with the extension of life expectancy, the demand of the middle-aged and elderly for related medical and elderly care services will continue to show an upward trend in the future.

Higher medical service satisfaction can enhance the middle-aged and elderly’s acceptance of medical and elderly care services, which reflects their demand for high-quality services and the important role of medical service levels in medical services. Therefore, to actively cope with the aging population, medical institutions can improve the middle-aged and elderly’s satisfaction with medical services by improving the quality of doctors’ technical skills [35], enhancing the effect of medical services, and promoting the accessibility and convenience of services, in order to improve the elderly’s acceptance rate of medical and elderly care services.

In addition, by comparing the acceptance differences between medical and elderly care services, it can be found that a regular physical examination is the most accepted service for the middle-aged and elderly. Middle-aged and elderly people who receive two or more medical and elderly care services choose the following item combination most frequently: “Regular physical examination + Onsite visit”, and “Regular physical examination + Entertainment services” is also selected by many old people. This shows that the two services of regular physical examinations and onsite visits are often chosen by the middle-aged and elderly, and the needs for these two services have a certain synergy.

### 4.3. Limitations

This study, however, also has some limitations: 1. This study is a cross-sectional study, and its causal inference ability is limited. 2. Deleting some samples with missing variable values may cause unknown bias in the results of this article. 3. Some questions are answered through participants’ recollections, which can lead to recall bias. 4. Due to the limited information on the CHARLS questionnaire, this study lacks the analysis of the reasons why the elderly choose to accept or not accept different medical and elderly care services. Therefore, these aspects can be supplemented and improved in future studies, in order to promote the further development of this topic.

## 5. Conclusions

This study explored the acceptance of home/community medical services by middle-aged and elderly people in China and the influencing factors. About 20.12% of middle-aged and elderly people in China have received home/community medical and elderly care services. Among them, a regular physical examination is the service with the highest acceptance rate for middle-aged and elderly people (16.87%). For the acceptance structure, the two services of regular physical examinations and onsite visits are often chosen by the elderly. In addition, the elderly aged 65–74 who have high satisfaction with local medical services are more likely to accept home/community-based medical and elderly care services.

In theory, this study promotes the study of medical and elderly care services. Many studies have explored the elderly’s desire and preference for medical and elderly care services, while this study focuses on their actual acceptance of related medical and elderly care services, and makes an integration and comparative analysis, thus revealing the acceptance rates of various medical and elderly care services and their combinations by middle-aged and elderly people and their influencing mechanisms.

In practice, the government should make greater efforts to publicize medical and elderly care services, increase the knowledge of middle-aged and elderly people about medical and elderly care services, and encourage them to receive medical and care services. In addition, the government should gradually improve this population’s satisfaction with local medical services, and focus on the needs of middle-aged and elderly people aged 65–74 for various medical services, in order to enhance the acceptance rate of medical and elderly care services.

## Figures and Tables

**Table 1:** Receiving rate of the home/community-based medical and elderly care services among middle-aged and elderly Chinese, 2018. (N, %).

Factors	All Samples (N = 9719)	1. Comprehensive Aged Care Services		2. Regular Physical Examination		3. Onsite Visits		4. Health Management		5. Entertainment	
		N ^1^ (%)	*p * ^2^	N ^1^ (%)	*p * ^2^	N ^1^ (%)	*p * ^2^	N ^1^ (%)	*p * ^2^	N ^1^ (%)	*p * ^2^
**Sex**											
Male	4787 (49.25)	57 (1.19)		791 (16.52)		157 (3.28)		88 (1.84)		129 (2.69)	
Female	4932 (50.75)	50 (1.01)	0.403	849 (17.21)	0.364	166 (3.37)	0.813	68 (1.38)	0.071	116 (2.35)	0.281
**Age group**											
45–64	3095 (31.84)	19 (0.61)		284 (9.18)		70 (2.26)		31 (1.00)		73 (2.36)	
65–74	4699 (48.35)	59 (1.26)	0.005 **	972 (20.69)	<0.001 ***	168 (3.58)	<0.001 ***	87 (1.85)	0.005 **	131 (2.79)	0.236
75+	1925 (19.81)	29 (1.51)		384 (19.95)		85 (4.42)		38 (1.97)		41 (2.13)	
**Marital status**											
Married	7746 (79.70)	76 (0.98)	0.025 *	1270 (16.40)	0.013 *	244 (3.15)	0.059	118 (1.52)	0.204	192 (2.48)	0.600
Others ^3^	1973 (20.30)	31 (1.57)		370 (18.75)		79 (4.00)		38 (1.93)		53 (2.69)	
**Hukou**											
Rural resident	7483 (76.99)	76 (1.02)	0.140	1192 (15.93)	<0.001 ***	267 (3.57)	0.014 *	110 (1.47)	0.053	167 (2.23)	0.001 **
Urban resident	2236 (23.01)	31 (1.39)		448 (20.04)		56 (2.50)		46 (2.06)		78 (3.49)	
**Education**											
No formal	2875 (29.58)	30 (1.04)		427 (14.85)		122 (4.24)		32 (1.11)		45 (1.57)	
Elementary school	4375 (45.01)	48 (1.10)	0.943	835 (19.09)	<0.001 ***	139 (3.18)	0.005 **	69 (1.58)	0.013 *	108 (2.47)	<0.001 ***
Middle school	1537 (15.81)	17 (1.11)		237 (15.42)		40 (2.60)		36 (2.34)		57 (3.71)	
High school and above	932 (9.59)	12 (1.29)		141 (15.13)		22 (2.36)		19 (2.04)		35 (3.76)	
**Income**											
Low	1811 (18.63)	23 (1.27)		217 (11.98)		60(3.31)		27 (1.49)		45 (2.48)	
Middle	5017 (51.62)	52 (1.04)	0.716	888 (17.70)	<0.001 ***	203 (4.05)	<0.001 ***	65 (1.30)	0.007 **	101 (2.01)	0.001 **
High	2891 (29.75)	32 (1.11)		535 (18.51)		60 (2.08)		64 (2.21)		99 (3.42)	
**Pension**											
No	935 (9.62)	10 (1.07)	0.923	147 (15.72)	0.322	36 (3.85)	0.344	9 (0.96)	0.100	13 (1.39)	0.020 *
Yes	8784 (90.38)	97 (1.10)		1493 (17.00)		287 (3.27)		147 (1.67)		232 (2.64)	
**Health insurance**											
No	281 (2.89)	1 (0.36)	0.225	24 (8.54)	<0.001 ***	14 (4.98)	0.115	3 (1.07)	0.467	3 (1.07)	0.115
Yes	9438 (97.11)	106 (1.12)		1616 (17.12)		309 (3.27)		153 (1.62)		242 (2.56)	
**Self-reported general health status**											
Bad	3031 (31.19)	28 (0.92)		504 (16.63)		111 (3.66)		37 (1.22)		46 (1.52)	
Fair	4666 (48.01)	56 (1.20)	0.517	798 (17.10)	0.844	147 (3.15)	0.451	73 (1.56)	0.013 *	125 (2.68)	<0.001 ***
Very good/good	2022 (20.80)	23 (1.14)		338 (16.72)		65 (3.21)		46 (2.27)		74 (3.66)	
**Smoke**											
No	7118 (73.24)	76 (1.07)	0.604	1253 (17.60)	0.001 **	241 (3.39)	0.570	119 (1.67)	0.387	182 (2.56)	0.708
Yes	2601 (26.76)	31 (1.19)		387 (14.88)		82 (3.15)		37 (1.42)		63 (2.42)	
**Alcohol drinking**											
No	6637 (68.29)	69 (1.04)	0.395	1116 (16.81)	0.819	228 (3.44)	0.366	94 (1.42)	0.030 *	137 (2.06)	<0.001 ***
Yes	3082 (31.71)	38 (1.23)		524 (17.00)		95 (3.08)		62 (2.01)		108 (3.50)	
**Satisfaction with local medical services**											
Dissatisfaction	1619 (16.66)	10 (0.62)		195 (12.04)		29 (1.79)		9 (0.56)		24 (1.48)	
Neutrality	4051 (41.68)	43 (1.06)	0.063	674 (16.64)	<0.001 ***	116 (2.86)	<0.001 ***	64 (1.58)	<0.001 ***	100 (2.47)	0.005
Satisfaction	4049 (41.66)	54 (1.33)		771 (19.04)		178 (4.40)		83 (2.05)		121 (2.99)	
**Chronic illness**											
None	1319 (13.57)	13 (0.99)		167 (12.66)		30 (2.27)		21 (1.59)		35 (2.65)	
1	2094 (21.55)	22 (1.05)	0.858	321 (15.33)	<0.001 ***	61 (2.91)	0.017 *	29 (1.38)	0.646	63 (3.01)	0.218
≥2	6306 (64.88)	72 (1.14)		1152 (18.27)		232 (3.68)		106 (1.68)		147 (2.33)	
**Economic welfare ^4^**	9719 (100)	107 (1.10)		1640 (16.87)		323 (3.32)		156 (1.61)		245 (2.52)	
**Medical welfare ^4^**	9719 (100)	107 (1.10)		1640 (16.87)		323 (3.32)		156 (1.61)		245 (2.52)	
**Social welfare ^4^**	9719 (100)	107 (1.10)		1640 (16.87)		323 (3.32)		156 (1.61)		245 (2.52)	

^1^ N (%) means the number of people receiving services (service acceptance rate %). ^2^ *p* value is the result of the chi-squared test describing the difference in the distribution of characteristics among people who receive services or not. * *p* < 0.05, ** *p* < 0.01, *** *p* < 0.001. ^3^ Includes divorced, separated, widowed, and single. ^4^ Source from the article of Wei Li et al. (see Reference [20] for details).

**Table 2:** Numbers of home/community-based medical elderly care services received by middle-aged and elderly Chinese, 2018. (N, %).

Factors	All Samples (N = 9719)	0	1	2	≥3	*p * ^1^
**Sex**						
Male	4787	3840 (80.22)	748 (15.63)	139 (2.90)	60 (1.25)	0.060
Female	4932	3924 (79.56)	818 (16.59)	152 (3.08)	38 (0.77)	
**Age group**						
45–64	3095	2710 (87.56)	317 (10.24)	49 (1.58)	19 (0.61)	
65–74	4699	3586 (76.31)	882 (18.77)	177 (3.77)	54 (1.15)	<0.001 ***
75+	1925	1468 (76.26)	367 (19.06)	65 (3.38)	25 (1.30)	
**Marital status**						
Married	7746	6240 (80.56)	1206 (15.57)	227 (2.93)	73 (0.94)	0.010 *
Others ^2^	1973	1524 (77.24)	360 (18.25)	64 (3.24)	25 (1.27)	
**Hukou**						
Rural resident	7483	6046 (80.80)	1153 (15.41)	214 (2.86)	70 (0.94)	0.001 **
Urban resident	2236	1718 (76.83)	413 (18.47)	77 (3.44)	28 (1.25)	
**Education**						
No formal	2875	2352 (81.81)	418 (14.54)	84 (2.92)	21 (0.73)	
Elementary school	4375	3408 (77.90)	792 (18.10)	131 (2.99)	44 (1.01)	<0.001 ***
Middle school	1537	1249 (81.26)	221 (14.38)	44 (2.86)	23 (1.50)	
High school and above	932	755 (81.01)	135 (14.48)	32 (3.43)	10 (1.07)	
**Income**						
Low	1811	1536 (84.82)	211 (11.65)	41 (2.26)	23 (1.27)	
Middle	5017	3961 (78.95)	853 (17.00)	164 (3.27)	39 (0.78)	<0.001 ***
High	2891	2267 (78.42)	502 (17.36)	86 (2.97)	36 (1.25)	
**Pension**						
No	935	754 (80.64)	157 (16.79)	17 (1.82)	7 (0.75)	0.120
Yes	8784	7010 (79.80)	1409 (16.04)	274 (3.12)	91 (1.04)	
**Health insurance**						
No	281	243 (86.48)	32 (11.39)	5 (1.78)	1 (0.36)	0.041 *
Yes	9438	7521 (79.69)	1534 (16.25)	286 (3.03)	97 (1.03)	
**Self-reported general health status**						
Bad	3031	2447 (80.73)	471 (15.54)	89 (2.94)	24 (0.79)	
Fair	4666	3728 (79.90)	743 (15.92)	144 (3.09)	51 (1.09)	0.437
Very good/good	2022	1589 (78.59)	352 (17.41)	58 (2.87)	23 (1.14)	
**Smoke**						
No	7118	5632 (79.12)	1191 (16.73)	223 (3.13)	72 (1.01)	0.019 *
Yes	2601	2132 (81.97)	375 (14.42)	68 (2.61)	26 (1.00)	
**Alcohol drinking**						
No	6637	5317 (80.11)	1069 (16.11)	195 (2.94)	56 (0.84)	0.113
Yes	3082	2447 (79.40)	497 (16.13)	96 (3.11)	42 (1.36)	
**Satisfaction with local medical services**						
Dissatisfaction	1619	1382 (85.36)	211 (13.03)	22 (1.36)	4 (0.25)	
Neutrality	4051	3253 (80.30)	647 (15.97)	113 (2.79)	38 (0.94)	<0.001 ***
Satisfaction	4049	3129 (77.28)	708 (17.49)	156 (3.85)	56 (1.38)	
**Chronic illness**						
No chronic disease	1319	1116 (84.61)	160 (12.13)	28 (2.12)	15 (1.14)	
One chronic disease	2094	1693 (80.85)	336 (16.05)	46 (2.20)	19 (0.91)	<0.001 ***
Two or more chronic diseases	6306	4955 (78.58)	1070 (16.97)	217 (3.44)	64 (1.01)	
**Economic welfare ^3^**	9719	7764 (79.88)	1566 (16.11)	291 (2.99)	98 (1.01)	
**Medical welfare ^3^**	9719	7764 (79.88)	1566 (16.11)	291 (2.99)	98 (1.01)	
**Social welfare ^3^**	9719	7764 (79.88)	1566 (16.11)	291 (2.99)	98 (1.01)	

^1^ *p* for the chi-squared test.* *p* < 0.05, ** *p* < 0.01, *** *p* < 0.001. ^2^ Includes divorced, separated, widowed, and single. ^3^ Source from the article of Wei Li et al. (see Reference [20] for details).

**Table 3:** Types of home/community-based medical and elderly care services received by middle-aged and elderly Chinese, 2018.

Rank	Number ^1^ = 1		Number = 2		Number = 3		Number = 4		Number = 5	
	Type ^2^	N (%)	Type	N (%)	Type	N (%)	Type	N (%)	Type	N (%)
1	2	1282 (81.86)	2 + 3	125 (42.96)	2 + 3 + 5	20 (26.67)	1 + 2 + 4 + 5	6 (35.29)	1 + 2+3 + 4 + 5	6 (100)
2	3	124 (7.92)	2 + 5	76 (26.12)	2 + 4 + 5	16 (21.33)	2 + 3 + 4 + 5	5 (29.41)		
3	5	92 (5.87)	2 + 4	51 (17.53)	1 + 2 + 3	11 (14.67)	1 + 2 + 3 + 4	3 (17.65)		
4	4	35 (2.23)	1 + 2	14 (4.81)	2 + 3 + 4	10 (13.33)	1 + 2 + 3 + 5	2 (11.76)		
5	1	33 (2.11)	1 + 4	6 (2.06)	1 + 2 + 5	8 (10.67)	1 + 3 + 4 + 5	1 (5.88)		
6			1 + 3	5 (1.72)	1 + 2 + 4	5 (6.67)				
7			3 + 4	4 (1.37)	1 + 3 + 4	2 (2.67)				
8			3 + 5	4 (1.37)	1 + 4 + 5	2 (2.67)				
9			1 + 5	3 (1.03)	3 + 4 + 5	1 (1.33)				
10			4 + 5	3 (1.03)	1 + 3 + 5	0 (0.00)				

^1^ Numbers of home and community care services received by elderly Chinese. ^2^ 1—Comprehensive aged care services; 2—Regular physical examination; 3—Onsite visits; 4—Health management; 5—Entertainment.

**Table 4:** Logistics regression model for influencing factors of receiving the home/community-based medical and elderly care services among middle-aged and elderly Chinese, 2018. (OR, 95% CI) ^1^.

Factors	1. Comprehensive Aged Care Services	2. Regular Physical Examination	3. Onsite Visits	4. Health Management	5. Entertainment
**Sex**					
Male	Reference	Reference	Reference	Reference	Reference
Female	0.88 (0.54–1.43)	1.10 (0.96–1.27)	0.83 (0.62–1.10)	0.86 (0.58–1.29)	1.21 (0.87–1.68)
**Age group**					
45–64	Reference	Reference	Reference	Reference	Reference
65–74	2.11 (1.23–3.61) ***	2.39 (2.06–2.76) ***	1.41 (1.05–1.89) *	2.03 (1.32–3.12) **	1.36 (1.01–1.85) *
75+	2.34 (1.26–4.38) ***	2.31 (1.93–2.76) ***	1.63 (1.15–2.30) **	2.16 (1.29–3.61) **	1.06 (0.70–1.61)
**Marital status**					
Married	Reference	Reference	Reference	Reference	Reference
Others ^2^	1.52 (0.97–2.40)	1.07 (0.93–1.23)	1.07 (0.81–1.41)	1.36 (0.91–2.02)	1.29 (0.92–1.79)
**Hukou**					
Rural resident	Reference	Reference	Reference	Reference	Reference
Urban resident	1.44 (0.82–2.54)	1.31 (1.12–1.54) **	1.04 (0.72–1.51)	0.89 (0.57–1.41)	1.17 (0.81–1.68)
**Education**					
No formal	Reference	Reference	Reference	Reference	Reference
Elementary school	1.03 (0.63–1.68)	1.43 (1.25–1.65) ***	0.81 (0.62–1.06)	1.45 (0.92–2.27)	1.57 (1.08–2.28) *
Middle school	1.12 (0.57–2.21)	1.24 (1.02–1.51)	0.81 (0.54–1.22)	2.24 (1.29–3.88) **	2.32 (1.48–3.64) ***
High school and above	1.25 (0.56–2.78)	1.14 (0.89–1.45)	0.85 (0.50–1.45)	1.79 (0.91–3.50)	2.13 (1.25–3.62) **
**Income**					
Low	Reference	Reference	Reference	Reference	Reference
Middle	0.69 (0.41–1.14)	1.34 (1.14–1.58)	1.15 (0.85–1.56)	0.69 (0.43–1.09)	0.70 (0.49–1.01)
High	0.58 (0.30–1.12)	1.28 (1.05–1.57)	0.66 (0.43–1.00)	1.07 (0.63–1.83)	0.83 (0.55–1.27)
**Pension**					
No	Reference	Reference	Reference	Reference	Reference
Yes	1.14 (0.58–2.24)	1.05 (0.87–1.28)	0.99 (0.68–1.44)	1.69 (0.84–3.39)	1.74 (0.97–3.10)
**Health insurance**					
No	Reference	Reference	Reference	Reference	Reference
Yes	3.72 (0.51–27.23)	2.09 (1.36–3.22) **	0.73 (0.41–1.29)	1.24 (0.39–4.02)	1.88 (0.59–6.00)
**Self-reported general health status**					
Bad	Reference	Reference	Reference	Reference	Reference
Fair	1.32 (0.82–2.13)	1.04 (0.91–1.19)	1.01 (0.78–1.31)	1.15 (0.76–1.75)	1.49 (1.05–2.13) *
Very good/good	1.28 (0.70–2.33)	1.09 (0.92–1.29)	1.09 (0.78–1.53)	1.68 (1.04–2.71) *	1.93 (1.28–2.91) **
**Smoke**					
No	Reference	Reference	Reference	Reference	Reference
Yes	1.11 (0.68–1.80)	0.91 (0.79–1.05)	1.00 (0.75–1.35)	0.73 (0.48–1.11)	0.83 (0.60–1.16)
**Alcohol drinking**					
No	Reference	Reference	Reference	Reference	Reference
Yes	1.20 (0.77–1.87)	1.07 (0.94–1.22)	0.99 (0.76–1.30)	1.30 (0.91–1.87)	1.66 (1.25–2.22) **
**Satisfaction with local medical services**					
Dissatisfaction	Reference	Reference	Reference	Reference	Reference
Neutrality	1.73 (0.86–3.46)	1.45 (1.22–1.73) ***	1.70 (1.12–2.57) *	2.88 (1.42–5.81) **	1.58 (1.00–2.49) *
Satisfaction	2.17 (1.09–4.31) *	1.72 (1.44–2.04) ***	2.43 (1.63–3.63) ***	3.79 (1.89–7.62) ***	2.07 (1.32–3.24) **
**Chronic illness**					
No chronic disease	Reference	Reference	Reference	Reference	Reference
One chronic disease	1.09 (0.55–2.19)	1.21 (0.99–1.49)	1.28 (0.82–2.00)	0.96 (0.54–1.71)	1.27 (0.83–1.94)
Two or more chronic diseases	1.24 (0.66–2.32)	1.49 (1.24–1.80) ***	1.73 (1.15–2.59) **	1.29 (0.78–2.13)	1.12 (0.75–1.67)
**Economic welfare ^3^**	0.98 (0.68–1.40)	1.13 (1.03–1.25) **	0.81 (0.63–1.03)	0.93 (0.69–1.25)	1.11 (0.90–1.38)
**Medical welfare ^3^**	1.15 (0.87–1.51)	0.77 (0.71–0.85) ***	0.94 (0.79–1.12)	0.78 (0.60–1.01)	1.04 (0.86–1.27)
**Social welfare ^3^**	0.99 (0.78–1.26)	1.10 (1.03–1.17) **	1.14 (1.00–1.31)	0.95 (0.76–1.17)	1.12 (0.97–1.29)

^1^ *p* for logistics regression. * *p* < 0.05, ** *p* < 0.01, *** *p* < 0.001. ^2^ Includes divorced, separated, widowed, and single. ^3^ Source from the article of Wei Li et al. (see Reference [20] for details).

## Data Availability

The datasets 2018 CHARLS for this study are a public database, and can be found on the website http://charls.pku.edu.cn/en/ (accessed on 1 August 2023).
